# A Novel Site-Specific Integration System for Genetic Modification of *Aspergillus flavus*

**DOI:** 10.1534/g3.119.400699

**Published:** 2019-12-09

**Authors:** Fang Tao, Kai Zhao, Qianqian Zhao, Fangzhi Xiang, Guomin Han

**Affiliations:** * School of Life Sciences, Anhui Agricultural University, Hefei 230036, P, R, China and; † The National Engineering Laboratory of Crop Stress Resistance Breeding, Anhui Agricultural University, Hefei, China

**Keywords:** site-specific integration, *sdh2*-locus, *Aspergillus flavus*

## Abstract

*Aspergillus flavus* is a fungus that produces aflatoxin B1, one of the most carcinogenic secondary metabolites. Understanding the regulation mechanism of aflatoxin biosynthesis in this fungus requires precise methods for genomic integration of mutant alleles. To avoid the disadvantage of DNA integration into the genome by non-homologous or ectopic recombination, we developed a novel strategy for site-specific integration of foreign DNA by using a carboxin-resistant *sdh2^R^* allele (His 249 Leu). Our results demonstrated that the transformants were generated with a high efficiency (>96%) of correct integration into the *sdh2*-lcus of the genome of *A. flavus* NRRL 3357. The advantage of this method is that introduction of the eGFP expression cassette into the *sdh2*-locus had little effect on fungal growth and virulence while also being rapid and efficient. This system will be a valuable tool for genetic manipulation in *A. flavus*. To the best of our knowledge, this is the first report on the efficient site-specific integration at the *sdh2*-locus in the genome of *Aspergillus*.

Homologous recombination is a powerful tool for generating stable gene replacement mutants in fungal genetic transformation systems, however, most exogenous DNA sequences are integrated into the genome by non-homologous, ectopic recombination. Such random ectopic integration can often disrupt the structure of non-target genes resulting in unpredictable effects on gene expression. In practice, the low frequency of homologous recombination makes the construction of gene disruptants in fungi a laborious process (Takahashi *et al.* 2006). Deletion of certain repair genes from the nonhomologous end joining pathway, *e.g.*, *ku70*, *ku80*, greatly enhanced the gene deletion frequency (Chang *et al.* 2010; Koh *et al.* 2014). However, while use of such *ku70*/*ku80* deleted mutants as recipient strains increases gene-targeting frequency, a second round of complementation with native *ku70*/*ku80* genes is needed before investigation of the deletion phenotype for the gene of interest. This additional transformation step is costly and time consuming, a disadvantage that can be surmounted by site-specific integration of genetic constructs without disrupting other regions of fungal genomic DNA (Weld *et al.* 2006).

Mitochondrial succinate dehydrogenase (SDH) is a key enzyme in the tricarboxylic acid cycle. Another name of the enzyme is succinate: ubiquinone reductase (SQR) as it catalyzes the coupled reactions of succinate oxidation to fumarate and the reduction of ubiquinone to ubiquinole. The SDH complex normally consists of a soluble catalytic heterodimer containing a flavoprotein subunit (SdhA/SdhFp), an iron–sulfur protein subunit (SdhB/Sdi1/Sdh2/Ip), and two hydrophobic polypeptides (SdhC and SdhD). Structural analyses have revealed that the ubiquinone binding site is composed of residues that are contributed from each of the SdhB, SdhC and SdhD subunits. The systemic fungicide carboxin inhibits the enzyme activity of succinate dehydrogenase by binding to the same site as ubiquinone (Horsefield *et al.* 2006; Huang *et al.* 2006). A single histidine-to-leucine point mutation in the third cysteine-rich cluster of the SdhB subunit confers resistance to carboxin and was first used as dominant selectable marker in *Ustilago maydis* (Broomfield and Hargreaves 1992; Keon *et al.* 1991). This valuable selection marker was then used in several other fungi, such as *Zymoseptoria tritici* (Kilaru *et al.* 2015), *Magnaporthe oryzae* (Guo *et al.* 2016), *Mortierella alpine* (Ando *et al.* 2009), *Aspergillus oryzae* (Shima *et al.* 2009), and a few mushrooms (Herzog *et al.* 2019; Shang *et al.* 2018). Although point mutations in either SdhC or SdhD were also shown to confer carboxin resistance (Ito *et al.* 2004), the SdhB-type mutations exhibited the strongest resistance (Shima *et al.* 2009).

*A. flavus* often produce aflatoxins with highly severe toxicity to human and animals (Han *et al.* 2019). Genetic manipulation, via gene knock-in or knock-out schemes, is an effective method to understand the regulation mechanisms of aflatoxin biosynthesis. Established methods for the transformation of *A. flavus* NRRL 3357 rely on either Polyethyleneglycol (PEG)-mediated protoplast transformation or *Agrobacterium tumefaciens*-mediated transformation (ATMT) (Han *et al.* 2018; He *et al.* 2007). Although the recently established ATMT is more efficient than the PEG-mediated transformation method, foreign DNA sequences are still integrated randomly into the genome (Han *et al.* 2018). In the present study, based on the ATMT system, we used the *sdh2* gene to create a specific locus as a “soft-landing” site for single copy insertions into the *A. flavus* genome. The pFC-*eGFP* vector designed for site-specific homologous recombination and harboring the *sdh2* mutant was established in *A. flavus* via ATMT. The coding region for eGFP was used as the foreign DNA sequence and carboxin as selection marker in this system. Here, we also provide a yeast recombination cloning strategy to assemble DNA fragments in a single step for high throughput vector generation.

To our knowledge, this is the first report utilizing the robustness of the yeast recombination cloning approach in combination with efficient site-specific integration into the genome of an *Aspergillus* species. This system will be a powerful tool for high throughput functional genomics studies in *A. flavus*.

## Materials and Methods

### Strains and culture conditions

*Escherichia coli* strain DH5α was used for vector cloning and plasmid maintenance. *A. tumefaciens* strain AGL-1 was used for *A. flavus* transformation. *E. coli* and *A. tumefaciens* strains were grown in DYT media (tryptone, 16 g/l; yeast extract, 10 g/l; NaCl, 5 g/l; with 15 g/l agar added for preparing the plates) at 37° and 28° respectively.

*Saccharomyces cerevisiae* strain FY834 (*MATα*; *hisΔ200*; *ura3-52*; *leu2Δ1*; *lys2Δ202*) was used for recombination-based cloning (Kilaru *et al.* 2015). The strain was refreshed on YPD agar medium (yeast extract, 10 g/l; glucose, 20 g/l, peptone, 20 g/l) at 28° for 48 h, and then used for preparing competent cells using the PEG/LiAC method (Gietz and Schiestl 2007). Yeast transformants were selected on Sc-U medium (yeast nitrogen base, 1.7 g/l; ammonium sulfate, 5 g/l, casein hydrolysate, 5 g/l; adenine hemisulfate salt, 20 mg/l; glucose, 20 g/l).

The *A. flavus* wild-type isolate NRRL 3357 was used as the recipient strain for fungal genetic transformation. The isolate was grown at 30°on potato dextrose agar (PDA, Difco) plates in the dark for 7 days. Fresh conidia were then harvested and used for transformation experiments.

For the carboxin sensitivity test, fungal spores were point inoculated onto MM [Czapek-Dox Broth (Difco) + 1.5% agar] plates with different concentrations of carboxin and cultured at 30° for 3 days. Wickerham medium (Chang *et al.* 2017) was used for observation of sclerotium formation. Aflatoxin analysis was carried out on strains grown on YES medium (20 g/l yeast extract, 150 g/l sucrose, 15 g/l agar).

### Construction of a yeast-escherichia-agrobacterium shuttle vector pUM

The plasmid pUM is a vector built on the framework of the binary vector pDHt (Han *et al.* 2018). To construct the pUM vector, a 2.9-kb *URA3-2*micron (*μ*) origin fragment was amplified from the pYES2 plasmid (Invitrogen, USA) with primers P97/P98 (Table 1), and then inserted into the *Sac* II site of pDHt.

**Table 1:** Primers used in this study

Primer name	Primer sequences (5′-3′)*^a^*	Remark
P101	*TGGCAGGATATATTGTGGTGTAAACAAATT*AGGGTATCTGTGGAAGCTGTG	*sdh2* left flank 592 bp *sdh2* coding sequence
P102	CAGTTAAGAATGGTGAGGCAACG	
P103	CTACCGTTGCCTCACCATTC	103 bp of 3′ end *sdh2* gene and 335 bp downstream of the *sdh2* gene
P104	CAGTTACGGAACAAAGGTCG	
P21	*CCGATTTTGCCGACCTTTGTTCCGTAACTG*ATTGCCTCTTTGCCTCCTAACAG	*tef1* promoter
P22	*GGTGAACAGCTCCTCGCCCTTGCTCACCAT*TTTGAAGGTGGTGCGAACTTTGTAG	
P31	ATGGTGAGCAAGGGCGAGGAG	*eGFP*
P32	TTACTTGTACAGCTCGTCCATG	
P41	*ACTCTCGGCATGGACGAGCTGTACAAGTAA*GGGATCCACTTAACGTTACTG	*trpC* terminator
P42	*TCCGGCGGGCCGATCCATAACCTTCACATG*TCGAGTGGAGATGTGGAGTG	
P105	CATGTGAAGGTTATGGATCG	right flank of *sdh2*
P106	*TAAACGCTCTTTTCTCTTAGGTTTACCCGC*TTGTCTGGGTCGGAGTTGCTCTG	
P97	CCGCGGGGAACAACACTCAACCCTA	*URA3-2*micron (*μ*) origin fragment amplification
P98	CCGCGGTTCGATGTAACCCACTCG	
P100	ATGGCTGCTCTTCGCTCAACCTC	Genomic PCR amplification
P32	TTACTTGTACAGCTCGTCCATG	
P105	CATGTGAAGGTTATGGATCG	DNA hybridization probes amplification
P107	TCTGGGTCGGAGTTGCTCTG	

a Italics indicate part of the primer that is complementary with another DNA fragment, to be ligated by homologous recombination in *S. cerevisiae*. Underlined letters indicated enzyme digestion site of *Sac* II.

### Construction of targeted ectopic integration vector pFC-eGFP

The pFC-*eGFP* vector was generated by *in vivo* recombination in the yeast strain FY834 following published procedures (Guo *et al.* 2016). To facilitate assembly of the DNA fragments in yeast, primers were designed with 30 bp DNA sequences homologous to the upstream and downstream of regions of the adjacent fragments to be combined together. The resulting pFC-*eGFP* vector contains *eGFP* under the control of the *A. flavus tef1* promoter and the *A. nidulans* trpC transcription terminator for integration into the *sdh2* locus by using carboxin as a selection agent. A 9735 bp fragment of pUM (digested with *Xho* I and *Eco*R I), a 592 bp region of the *sdh2* coding sequence (amplified with P101/P102), a point-mutated (H249L) 438 bp fragment containing 103 bp of the 3′ end of the *sdh2* gene, and a 335 bp region downstream of the *sdh2* gene (amplified with P103/P104), a 838 bp portion of the *tef1* promoter (amplified with P21/P22), a 720 bp coding sequence of *eGFP* (amplified with P31/P32), a 771 bp *trpC* terminator (amplified with P41/P42), and a 1074 bp fragment covering the right flank of the *sdh2* gene (amplified with P105/P106) were designed to have overlapping homologous sequences and assembled according to Figure 3A. All above DNA fragments were transformed into the FY834 competent yeast cells following a small-scale yeast transformation protocol in the pYES2 user manual (Invitrogen, USA) and selected on Sc-U medium. The homologous recombination plasmid products were purified using the TIANprep yeast plasmid DNA kit (DP112, Tiangen Biotech, China), and then transformed into *E. coli* DH5α competent cells. The DNA sequence of the final assembled pFC-*eGFP* plasmid was confirmed by PCR and DNA sequencing, after which it was transformed into the AGL-1 strain using the freeze/thaw shock transformation method. Primers used in this study were listed in Table 1.

### A. flavus transformation

*A. tumefaciens*-mediated transformation of *A. flavus* was performed as described previously (Han *et al.* 2018), with some modification. The *A. tumefaciens* AGL-1 strain containing the pFC-*eGFP* plasmid was grown in 10 ml DYT medium supplemented with rifampicin (20 mg/ml) and kanamycin (50 μg/ml) overnight at 28° with shaking at 200 rpm. The overnight culture was then diluted to an OD_600_ of 0.15 with induction medium (IM) and grown at 28° with shaking at 200 rpm until reaching an OD_600_ 0.35-0.4. The *A. tumefaciens* cultures were then mixed with an equal volume of *A. flavus* conidial suspensions (2×10^6^ spores/ml), and subsequently 200 μl of the mixed cultures were plated onto cellulose nitrate membranes (0.45 μm pore, Sartorius Biotech, Goettingen, Germany) placed on co-cultivation medium and grown at 22° for 48 h. The cellulose nitrate membranes were then transferred to selection medium containing 300 μg/ml cefotaxime (Sangon Biotech, China), 60 μg/ml streptomycin (Bomei, China) and 50 μg/ml carboxin (45371, Sigma-Aldrich, Germany) and incubated at 28° in the dark until colonies appeared. The individual colonies were transferred to selection medium with the appropriate antibiotics, as described above, and grown at 28° for 3-4 days.

### Molecular analysis of transformants

Isolation of single spores from each transformant was achieved by spreading the diluted spore suspension onto PDA plates and incubation at 30° for 1-2 days. Individual conidia were then transferred to new PDA plates and incubated at 30° for a further 7 days. Genomic DNA was obtained by using a modified CTAB method (Han *et al*. 2018) and used for PCR amplification performed using primers P100/P32 to validate the specific integration of the vector pFC-*eGFP* into the *sdh2* locus of *A. flavus*.

For Southern blot detection, genomic DNA isolated from the transformants was digested with the *Hin*d III and separated by electrophoresis in a 1.0% agarose gel and capillary transferred onto a Hybond-N^+^ membrane (GE Healthcare). A specific probe was generated with by PCR with primers P105/107, labeled with digoxigenin-dUTP, and hybridized with the membrane according to the instruction (11585614910, Roche, Germany). The wild-type *A. flavus* NRRL 3357 genomic DNA was used as the negative control.

### Microscopy

The expression of GFP in *A. flavus* transformants was analyzed using a Leica DM5000 B fluorescence microscope (Leica, Germany). The selected transformants were incubated on PDA plates at 30° for 2-5 days after which spores, mycelia and conidiophores were collected for fluorescence analysis. The wild-type strain NRRL 3357 was used as negative control.

### Conidial and sclerotial production

For quantitative comparison of the production of conidia, an aliquot of conidial suspension (10^4^ spores) was point inoculated onto the center of a PDA plate. Cultures were grown at 30° for 5 days. Conidia were washed off the agar plates using 0.01% Triton X-100 solution and counted in a hemocytometer.

For sclerotial analysis, conidial suspensions (10^4^ spores) were spread on Wickerham medium agar plates and incubated in darkness at 37° for 10 days. Conidia were washed off the plates using 0.01%Triton X-100 solution, and remaining sclerotia were counted under a microscope.

### Quantitative determination of aflatoxin B1 production by HPLC

The method for quantitative comparison of the production of aflatoxin B1 was conducted as previously described with some modification (Han *et al.* 2018). A conidial suspension (2×10^4^ spores) was seeded centrally onto sterile cellophane sheets that were placed over a YES agar plate and incubated at 30° for 5 days. The fungal biomass was scraped from the plates, and extracted by incubation with 5 ml of methanol at room temperature with shaking at 150 rpm for 30 min. The supernatant was collected by centrifugation at 3,000 g and filtered through a syringe filter (RC 0.22 μm, Alltech, Deerfield, IL). The presence of aflatoxin B1 was determined by HPLC with fluorescence detection, using a Waters 600 series HPLC equipped with a 600 pump, a 2707 autosampler and a 600 column thermostat set at 30°. Detection was performed using a 2475 Multi λ fluorescence detector set at 365 nm (λex) and 465 nm (λem), with a Waters Empower Windows xp operating system (Waters, Milford, MA, USA). The analytical column was a Luna 3u C18 (2) (150×4.6 mm, 3 μm) (Phenomenex, Torrance, CA, USA) preceded by a SecurityGuard TM precolumn (C18, 4×3.0 mm, Phenomenex). The mobile phase consisted of methanol: water (55:45), eluted at a flow rate of 0.6 ml/min, with 20 μl of filtered extract injected into the HPLC per run. Aflatoxin B1 production was measured in μg/g of mycelia.

### Kernel infection assays

Conidia of the *A. flavus* transformants were harvested from PDA plates and suspended in water and adjusted to a cell density of 4×10^6^ /ml. Undamaged maize seeds were surface-sterilized with 70% ethanol. Ten μl of spore suspension was dripped onto the embryo of the kernel which were then placed in 3.5 cm petri dishes and incubated in the dark at 30° for 7 days. High humidity (> 95% RH) was maintained.

### Data availability

Strains and plasmids used in this study are available upon request. The authors state that all data necessary for confirming the conclusions of the article are present within the article.

## Results

### Sensitivity of conidia germination to carboxin

**Figure 1:**
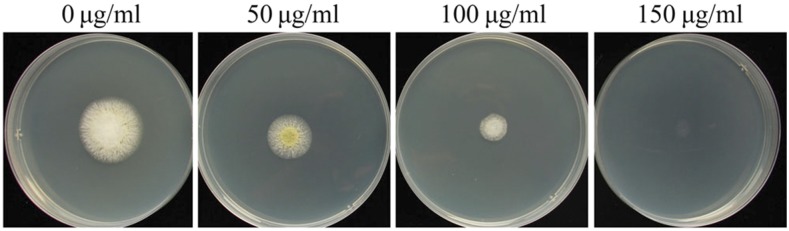
Sensitivity assay of *A. flavu*s mycelia to carboxin. *A. flavus* wild type strain NRRL 3357 cultured for 3 days on MM medium supplemented with various concentrations of carboxin.

In order to find a suitable concentration of antibiotic for screening transformants, the sensitivity of *A. flavus* wild type strain NRRL 3357 to carboxin was analyzed. The results showed that mycelial growth was completely inhibited in MM medium supplemented with 150 μg/ml carboxin (Figure 1).

### Identification of Sdh2 in A. flavus

**Figure 2:**
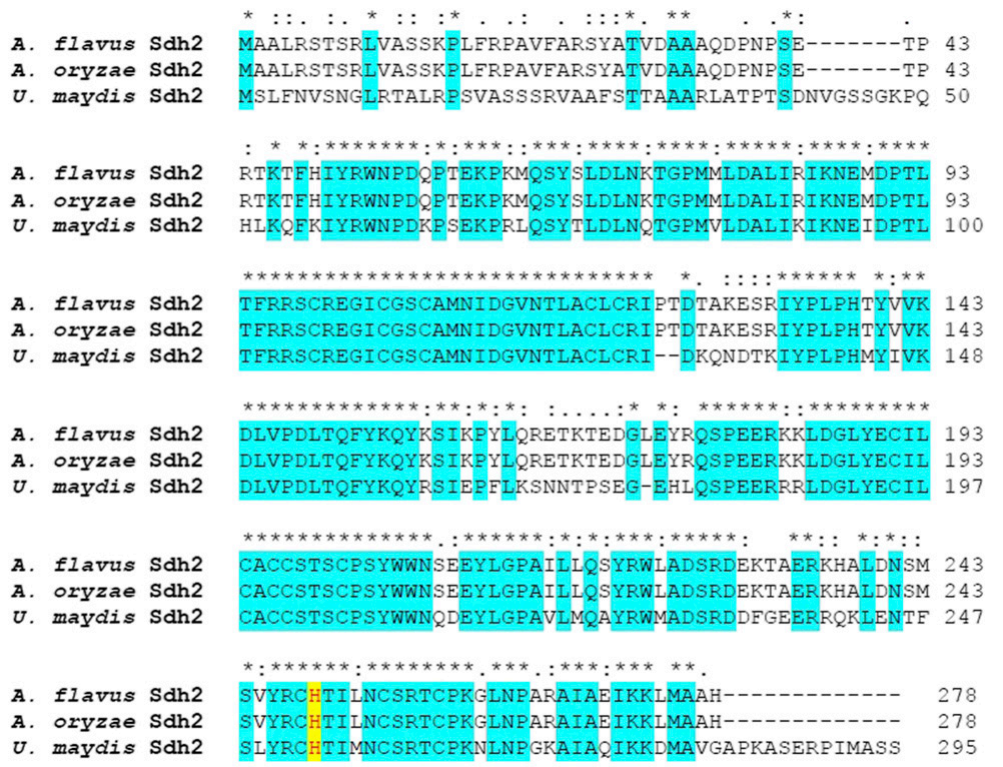
Comparison of the amino acid sequences of the succinate dehydrogenase subunit *Sdh2* of *A. flavus* (Accession No., EED45758), *A. oryzae* (Accession No., XP_001827486), and *U. maydis* (Accession No.,XP_011386878).

In *A. flavus* NRRL 3357, the iron-sulfur containing subunit of the succinate dehydrogenase enzyme encoded by the *sdh2* gene is a 278 amino acid protein (Accession number, EED45758) which shares 54.91% and 100% sequence similarities to the corresponding sequences in *U. maydis* (Accession No., XP_011386878) and *A. oryza*e RIB40 (accession number, XP_001827486), respectively (Figure 2). It has been proven that the substitution of the His (249) residue with a Leu in *A. oryzae* confers the highest resistance to the fungicide carboxin (Shima *et al.* 2009). The conserved histidine (249) in *Sdh2* should play a similar role in conferring resistance to the fungicide carboxin in *Aspergillus*.

### Construction of sdh2^R^ based vector

**Figure 3:**
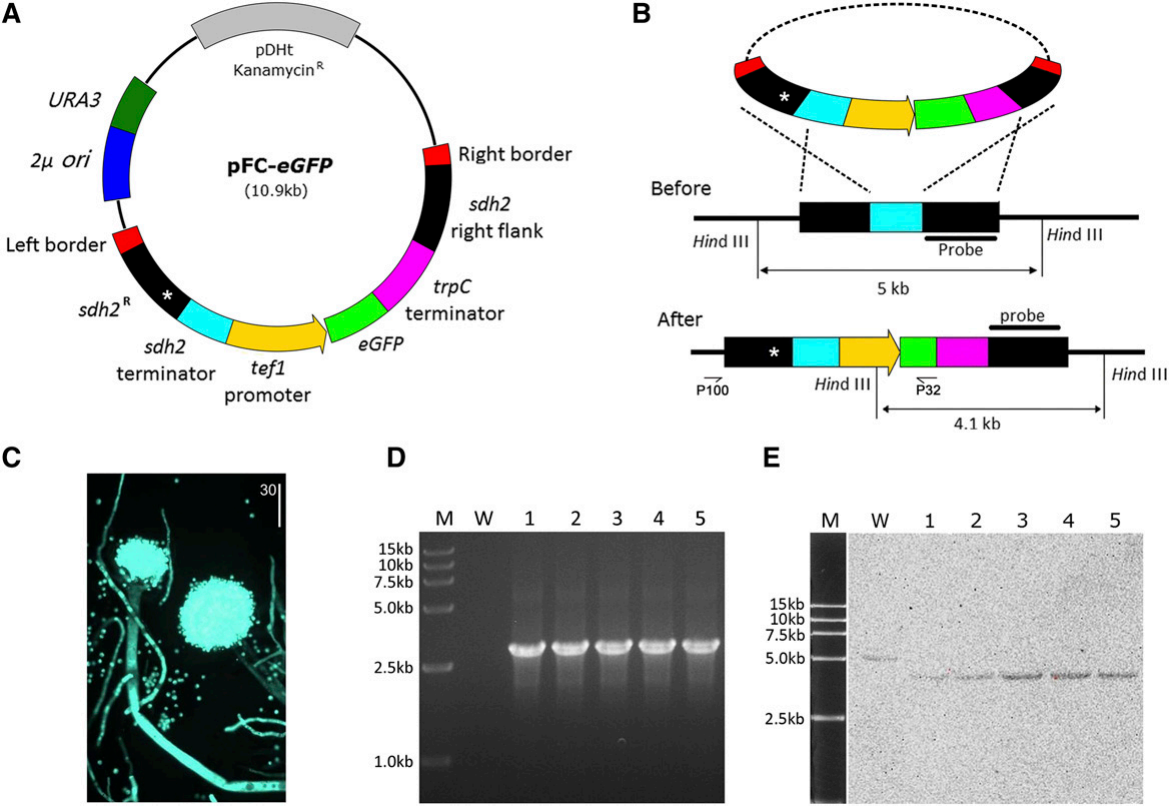
Construction of a vector for targeted integration into the *sdh2* locus in *A. flavus*. (A) Schematic drawing showing the organization of the pFC-*eGFP* vector. Expression of the fluorescent protein eGFP is under the control of the *A. flavus tef1* promoter and *A. nidulans trpC* terminator. *URA3* and *2μ* origin cassettes enable yeast recombination-based cloning in *S. cerevisiae*. After integration into the *sdh2* locus, a point mutation (indicated by an asterisk) in the succinate dehydrogenase encoding gene *sdh2* resulted in substitution of histidine to leucine (H249L), conferring carboxin resistance on the transformants. Note that fragments are not drawn to scale. For more accurate information on fragment sizes see main text. (B) Illustration of the integration process of pFC-*eGFP* into the native *sdh2* locus of *A. flavus*. Homologous recombination simultaneously inserts the carboxin-resistant *sdh2^R^* allele and eGFP cassette into the genome of *A. flavus*. (C) Image showing eGFP expression at different developmental stages of *A. flavus* after integration of pFC-*eGFP* into the *sdh2* locus. (D) The *sdh2^RG^* mutants were validated by PCR. M= DL15,000 DNA marker; W= wild type strain; 1-5= *sdh2^RG^* mutants. (E) Southern blot analysis of *sdh2^RG^* mutants. All carboxin-resistant transformants contained a single integration into the desired locus. M: DL15,000 DNA marker; W: wild type; 1-5: *sdh2^RG^* mutants.

A locus specific integration vector, pFC-*eGFP*, containing the mutant *sdh2* sequence was constructed as described in the methods. In this vector, RNA transcription of the *eGFP* gene is initiated by the *A. flavus tef1* promoter and terminated by the *A. nidulans trpC* terminator. To integrate the foreign DNA into the wild type *sdh2* locus, the *sdh2^R^* left and right flanks were introduced adjacent to the *eGFP* cassette. The most important characteristic feature of the *sdh2^R^* left flank is that it is made up of 695 bp from the 3′ end of the *sdh2* gene (the full-length gene is 1032 bp) containing the H249L point mutation and 335 bp of the *sdh2* terminator sequence (Figure 3A, left flank). The right flank contains the 1074 bp downstream regions of *sdh2*. The point mutation (H249L) in the *sdh2^R^* left flank will mutate the endogenous *sdh2* gene, conferring resistance to carboxin after integration by homologous recombination (Figure 3B).

In addition, a yeast recombination cassette consisting of *URA3* and a *2μ* ori was introduced into the vector. This method allows the multiple overlapping DNA fragments to be assembled in a single step by homologous recombination in yeast, avoiding laborious restriction/ligation-based cloning procedures.

### Targeted integration of the pFC-eGFP vector into the sdh2 locus

We transformed vector pFC-*eGFP* into the *A. flavu*s strain NRRL 3357 by *A. tumefaciens*-mediated transformation to generate *sdh2^R^* mutants expressing eGFP (*sdh2^RG^*). The transformants were selected on MM medium containing 150 μg/ml carboxin. To confirm the efficiency of targeted integration of pFC-*eGFP* into the native *sdh2* locus of *A. flavus*, genomic DNA was extracted from five randomly selected *sdh2^RG^* mutants. PCR analysis showed that a 2.9kb DNA fragment was obtained after amplification with primer pair P100/P32 in all mutants but not in wild type (Figure 3D). Southern blot assays identified a single band at the expected size (4.1kb) in all transformants, while a band of 5kb in size was identified in the wild type genomic DNA (Figure 3E). We found that 96% of transformants has a single copy integration into the *sdh2* locus (in total 52 transformants), and the remaining 4% of transformants has a second integration event via qPCR (Lu *et al.* 2014) (data not shown). Moreover, GFP fluorescence was observed at all developmental stages, including in the spores, mycelia and conidiophores from all analyzed *sdh2^RG^* mutants (Figure 3C).

### Phenotypes of carboxin-resistant transformants of A. flavus

**Figure 4:**
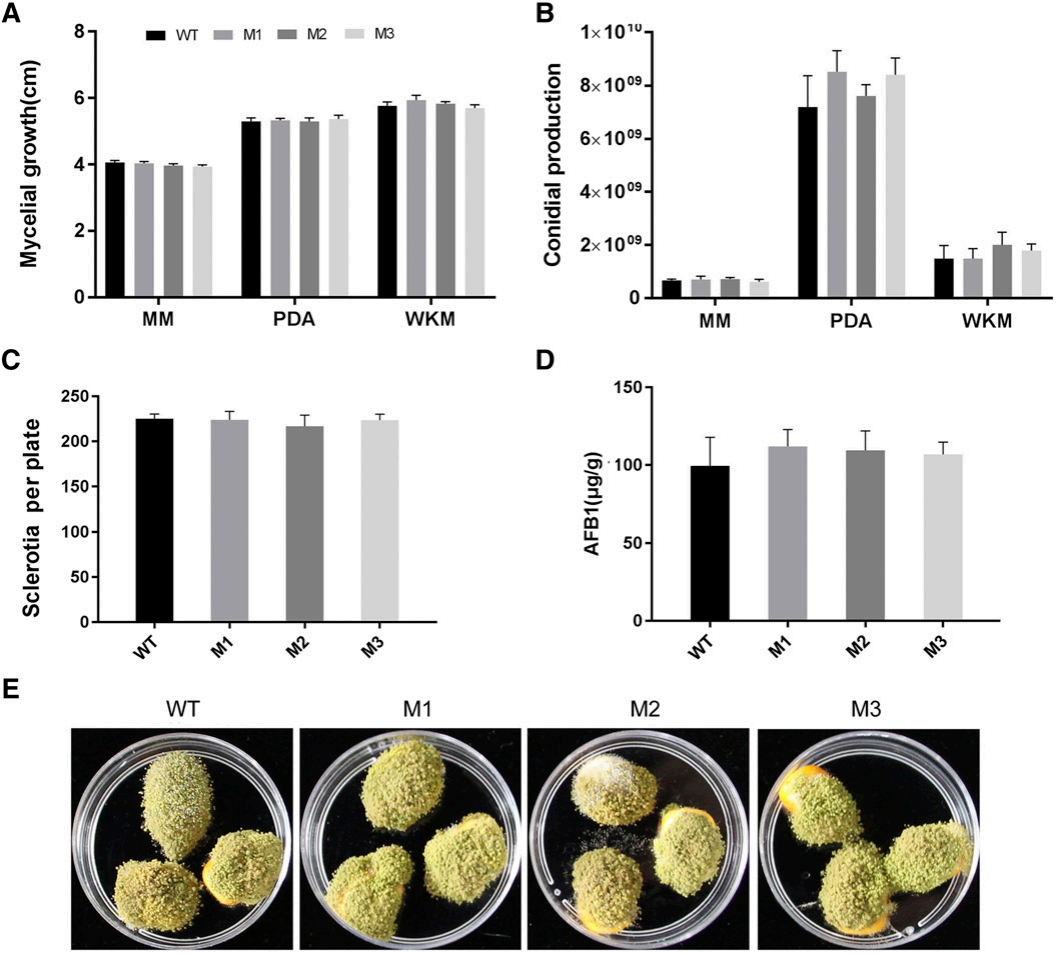
Phenotype analysis of wild-type and carboxin-resistant *A. flavus* strains. (A) Mycelial growth and (B) conidial production were detected after culturing on PDA plates. (C) Analysis of Sclerotia formation after culturing on WKM medium. (D) Fungal cultures on YES plates were extracted by methanol and AFB1 production quantified by HPLC analysis. (E) Infection rate was tested by inoculating spores onto maize kernels. WT: wild type; M: mutant.

To evaluate the effect of integration of pFC-*eGFP* into *sdh2* locus on *A. flavus* growth and development, we compared mycelial growth, as well as conidial and sclerotial production of carboxin-resistant mutants with that of the wild-type strain. Data displayed in Figure 4 shows that there were no significant differences observed between mutant and wild type strains. In addition, level of AFB1 production was also found to be similar in the two strains, indicating that integration of the vector into *sdh2* locus has little impact on aflatoxin synthesis. The infection rate of the mutants was investigated on maize kernels. No significant difference was observed between the wild-type strain and the mutants.

## Discussion

At present, considerable efforts have been made to overcome the difficulty of using non-homologous, ectopic recombination for DNA integration into the genomes of filamentous fungi. Indeed, work has focused on improving the frequency of gene targeting by homologous recombination, such as using split-marker- or deficient-NHEJ DNA repair pathway- strategies. However, split-marker technology is based on three crossover events, significantly reducing the transformation rate compared with classical recombination strategies based on a single crossover event (Aragona and Valente 2015; Wilson *et al.* 2010). Use of deficient-NHEJ repair pathway methods may generate unexpected effects in the reduction of DNA repair capacity (Emerson *et al.* 2018; Pannunzio *et al.* 2018).

Recently, a new strategy was developed based on specific integration at the site of a selectable marker gene in several filamentous fungi. Such suitable genes include *pyrG* in *A. niger* (van Hartingsveldt *et al.* 1987) and *A. awamori* (Gouka *et al.* 1995), ILV2 in *Magnaporthe oryzae* (Yang and Naqvi 2014), and *sdi1* in *Ustilago maydis*, *Zymoseptoria tritici* (Kilaru *et al.* 2015) and *M. oryzae* (Guo *et al.* 2016). The *pyrG* gene encodes orotidine-5′-monophosphate (OMP) decarboxylase, a key enzyme for biosynthesis of uridine/uracil which is required for fungal survival. It's a dominant selectable marker in many *Aspergillus* spp. While integration at the *pyrG* locus usually requires two steps. First, the *pyrG* gene in the recipient strain should be mutated to abrogate function, followed by integration of a wild-type *pyrG* gene at the mutated *pyrG* locus by homologous substitution (Gouka *et al.* 1995). However, compared with the laborious double integration method for insertion at the *pyrG* gene locus, integration at either *ILV2* or *sdi1* loci only requires one step, due to the single amino acid substitution in either gene that introduces drug resistance in recipient strain.

In this study, we devised a novel strategy for site-specific integration of foreign DNA at the *sdh2* gene locus in *A. flavus*. The vector pFC-*eGFP* harboring the mutant *sdh2^R^* allele containing a single amino acid substitution (His 249 Leu), was constructed via yeast recombination-based cloning for fungal transformation. The replacement of the native *sdh2* with the *sdh2^R^* allele confers resistance to carboxin in this fungus. The resulting transformants showed a high efficiency (>96%) of correct integration into the genome of *A. flavus*, with individual transformants having no detectable variations in the intensity GFP fluorescence. Crucially, introduction of the eGFP expression cassette at the *sdh2* locus did not discernably alter fungal growth and virulence. The *sdh2*-locus integration system will be a valuable tool not only for gene expression, but also for genetic complementation, promoter analyses, and protein cellular localization in *Aspergillus* species.

## Conclusion

In this study we developed an efficient carboxin-resistance recombination strategy for *sdh2* specific integration in *A. flavus*. This new strategy allows the precise integration of the DNA sequence of interest into the *sdh2*-locus without disturbing the expression of fungal genes. Additionally, the carboxin-resistance transformation does not influence fungal growth and virulence in any way. Thus, we have demonstrated the utility of the *sdh2* locus for targeted integration as a valuable tool for mutant strain generation in *A. flavus*.
